# Supplementary material for: Association between oral microbiome and seven types of cancers in East Asian population: a two-sample Mendelian randomization analysis
Source: Front Mol Biosci. 2023 Nov 21;10:1327893. doi: 10.3389/fmolb.2023.1327893 (PMC10702768; doi:10.3389/fmolb.2023.1327893)
Supplement: Supplementary file 1 [file DataSheet1.ZIP › Supplementary Materials/MR plots of saliva/saliva═╝/Prostate cancer/pheno.1102_to_prostate cancer_leave_one_out.pdf]

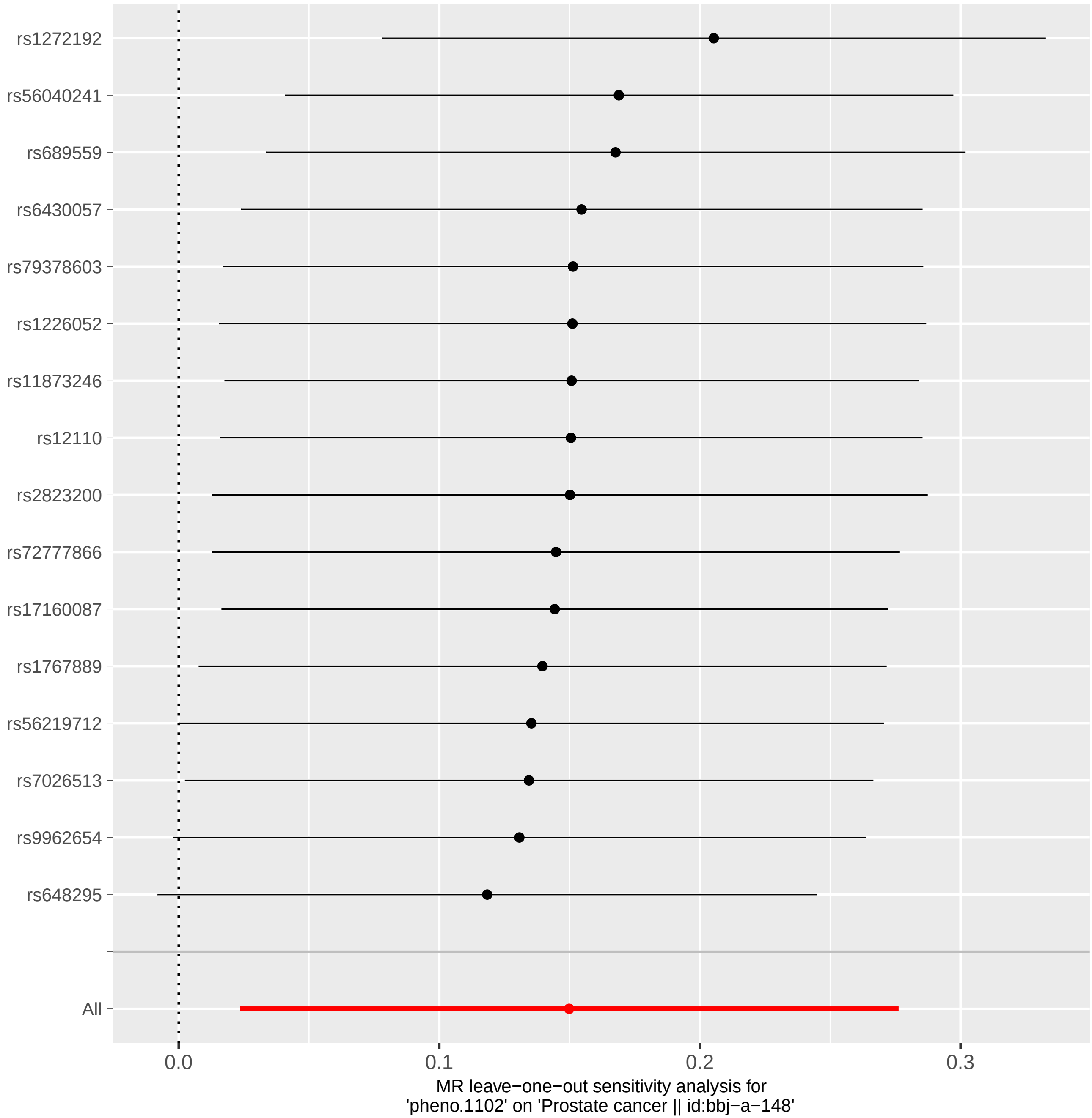

rs1272192
rs56040241
rs689559
rs6430057
rs79378603
rs1226052
rs11873246
rs12110
rs2823200
rs72777866
rs17160087
rs1767889
rs56219712
rs7026513
rs9962654
rs648295
All
0.0
0.1
0.2
0.3
MR leave−one−out sensitivity analysis for
'pheno.1102' on 'Prostate cancer || id:bbj−a−148'
